# Strong founder effect for *BRCA1* c.3629_3630delAG pathogenic variant in Chechen patients with breast or ovarian cancer

**DOI:** 10.1002/cam4.5159

**Published:** 2022-08-23

**Authors:** Anna P. Sokolenko, Luisa V. Sultanova, Ilya A. Stepanov, Alexandr A. Romanko, Aigul R. Venina, Tatiana N. Sokolova, Hedi S. Musayeva, Marina Ya. Tovgereeva, Mareta Kh. Magomedova, Khusein U. Akhmatkhanov, Elisa I. Vagapova, Elkhan A. Suleymanov, Elena V. Vasilyeva, Elvina Kh. Bakaeva, Ilya V. Bizin, Svetlana N. Aleksakhina, Evgeny N. Imyanitov

**Affiliations:** ^1^ Department of Tumor Growth Biology N.N. Petrov Institute of Oncology Saint‐Petersburg Russia; ^2^ Department of Medical Genetics St.‐Petersburg Pediatric Medical University Saint‐Petersburg Russia; ^3^ Chechen Republican Cancer Center Grozny Russia; ^4^ Department of Oncology I.I. Mechnikov North‐Western Medical University Saint‐Petersburg Russia

**Keywords:** *BRCA1* and *BRCA2* testing, breast cancer, Chechens, founder mutation, next‐generation sequencing, ovarian cancer

## Abstract

Coding sequences of *BRCA1, BRCA2, ATM, TP53,* and *PALB2* genes were analyzed in 68 consecutive Chechen patients with high‐grade serous ovarian cancer (HGSOC). Pathogenic *BRCA1/2* variants were identified in 15 (22%) out of 68 HGSOC cases. Nine out of ten patients with *BRCA1* pathogenic alleles carried the same deletion (c.3629_3630delAG), and three out of five *BRCA2* heterozygotes had Q3299X allele. The analysis of 49 consecutive patients with triple‐negative breast cancer (TNBC) revealed 3 (6%) additional *BRCA1* heterozygotes. All women with *BRCA1* c.3629_3630delAG allele also carried linked c.1067G>A (Q356R) single nucleotide polymorphism, indicating that this is a genuine founder variant but not a mutational hotspot. An *ATM* truncating allele was detected in one HGSOC patient. There were no women with *TP53* or *PALB2* germline alterations. Genetic analysis of non‐selected HGSOC patients is an efficient tool for the identification of ethnicity‐specific *BRCA1/2* pathogenic variants.

## Graphical abstract

Coding sequences of *BRCA1, BRCA2, ATM, TP53,* and *PALB2* were analyzed in 68 consequtive Chechen patients with high‐grade serous ovarian cancer and 79 patients with breast cancer. The recurrent *BRCA1* pathogenic variant was identified.

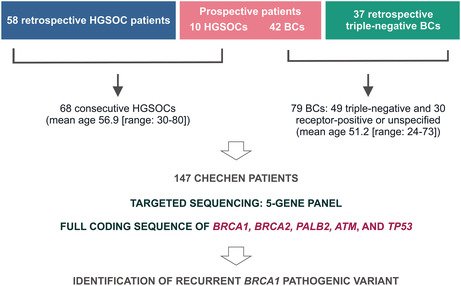

High‐risk pathogenic alleles make a significant contribution to cancer morbidity worldwide, accounting for approximately 5%–10% of breast cancer (BC) and 15%–20% of ovarian cancer (OC) incidence, respectively.^1^, ^2^ However, BC‐ and OC‐predisposing mutations across populations are, by definition, far from being distributed evenly. Each ethnic group originates from its own pool of ancestors, who were carriers of a unique spectrum of disease‐associated alleles. Therefore, national communities, which remained more or less biologically isolated over the past time, are usually characterized by distinct patterns of hereditary diseases and pathogenic variants.^3^, ^4^

Chechens are a large ethnic group residing mainly in the North Caucasus. The total number of Chechens is approximately 2 million, with the majority of them living in the Chechen Republic (Russia). Interethnic marriage is uncommon in the Chechen community.^5^, ^6^ Hence, there is a high probability that hereditary diseases observed in the Chechen population are attributed mainly to recurrent alleles. The primary aim of this study was the analysis of the spectrum of *BRCA1/2* germline mutations in patients of Chechen origin. The annual number of new BC and OC cases across the Chechen population is approximately 370 and 40, respectively.^7^ Given that only a subset of these patients belongs to clinical subgroups with hereditary features of cancer predisposition (e.g., early‐onset, family history, or disease bilaterality for BC patients, or high‐grade serous histology for OC patients), we estimated that it may take years to collect a substantial number of these patients prospectively. Several reports demonstrated that consecutive (i.e., unselected for family history or age at onset) series of high‐grade serous OC (HGSOC) were characterized by a high frequency of *BRCA1/2* mutations.^8^, ^9^, ^10^ We anticipated that the retrospective analysis of relatively small numbers of HGSOC patients would reveal whether *BRCA1/2* germline mutations persist in the Chechen population and whether the detection of *BRCA1/2* pathogenic variants can be simplified because of the existence of a founder effect.

The study included 68 consecutive HGSOC patients who underwent treatment in the Chechen Republican Cancer Center (Grozny, Russia) within the years 2011–2020. Normal archival tissues, which were obtained during medical procedures and were stored in the morphological archive of this hospital, served as a source for DNA (*n* = 58). In addition, we enrolled prospectively 52 consecutive patients (10 with HGSOC and 42 with BC) who were referred to this clinic between August 2020 and June 2021 and provided blood for genetic analysis. When a high frequency of *BRCA1* mutations in HGSOC was revealed, we added 37 archival triple‐negative BC cases to the study. The investigation was approved by the local Ethics Committee. Clinical characteristics are described in Table 1.

**TABLE 1:** Clinical description of the patient cohorts

Clinical feature	Patients
High‐grade serous ovarian carcinoma	*N* = 68^a^
Mean age at diagnosis (range)	56.9 (30–80)
Breast cancer	*N* = 79
Mean age at diagnosis	51.2 (24–73)
Triple‐negative carcinomas	49 (62%)
Receptor‐positive or unspecified	30 (38%)
Total	*N* = 147

*Note*: There was a numerical but statistically non‐significant difference between *BRCA1/2* mutation carriers and non‐carriers regarding mean age at diagnosis in both BC and OC cohorts (51.8 years vs. 43.6 years in sporadic and hereditary BCs, correspondingly [Student's *t*‐test, *p* = 0.24]; 57.6 years vs. 54.4 years in sporadic and hereditary OCs, correspondingly [Student's *t*‐test, *p* = 0.25]).

^a^ Including three patients with combination of breast and ovarian cancer.

DNA isolation from formalin‐fixed paraffin‐embedded (FFPE) samples was performed using Cobas DNA Sample Preparation Kit (Roche). DNA from blood lymphocytes was extracted by conventional protocol.^11^ Although the primary goal of the study was the analysis of *BRCA1/2* mutations, we added to the panel *PALB2*, *ATM*, and *TP53* genes considering their well‐established role in cancer predisposition. Genomic DNA (400 ng) was subjected to library preparation with KAPA HyperPlus Kit (Roche) according to manufacturer instructions. For FFPE samples, the adapter ligation time was extended to 20 h. Dual‐index libraries were used to pool up to 96 samples into one enrichment reaction. Pooled DNA libraries (1000 ng) were enriched with a custom panel of biotinylated probes covering coding sequences as well as exon‐intron boundaries and 5′‐ and 3′‐untranslated regions of the above‐mentioned genes. Due to the small size of the panel, two‐round overnight hybridization was performed. Enriched libraries were sequenced on the Illumina NextSeq 500 platform with the Mid Output Kit v2.5 reagents in a paired‐end mode for 150 cycles in both orientations. Mean depth‐of‐coverage reached ×1500 with 99.9% of bases read at least 100 times. The bioinformatic pipeline included standard steps, i.e., FASTQ files generation, quality assessment, and mapping of the obtained sequences to the hg19 genome using the BWA tool. Aligned reads were subjected to SNVs and indels calling with the HaplotypeCaller [GATK4]. Annotation was made with the SnpEff software instrument.

Next‐generation sequencing (NGS) analysis was performed for 68 consecutive Chechen patients with HGSOC. *BRCA1/2* pathogenic variants were observed in 15 (22%) out of 68 HGSOC cases (Table 2). 9 of 10 *BRCA1* heterozygotes carried the same allele (c.3629_3630delAG [rs80357589]), and one woman had c.5296delA nucleotide deletion. Greater genetic diversity was observed in HGSOC patients with *BRCA2* mutations (c.9895C>T [Q3299X; rs1555289997]: *n* = 3; c.5351dupA [rs80359507]: *n* = 1; c.7408_7409delTT [rs397507915]: *n* = 1). We also subjected to NGS testing 42 women with BC who were referred to the N.N. Petrov Institute of Oncology for diagnostic *BRCA1/2* testing. There was one carrier of *BRCA1* c.3629_3630delAG allele and two women with *BRCA1/2* splice‐site mutations (*BRCA1* c.5153‐2A>G [IVS18‐2A>G; rs786202545] and *BRCA2* c.9117G>A [P3039P; rs28897756], respectively). Given the high frequency of *BRCA1* pathogenic variants, we added to the analysis 37 consecutive retrospective patients with triple‐negative breast cancer (TNBC). Because *BRCA1* is specifically associated with the triple‐negative phenotype of breast cancer disease,^25^ we expected to increase the number of *BRCA1* heterozygotes. Two additional cases of *BRCA1* pathogenic variants (c.1338_1339delAG: *n* = 1; c.5296delA: *n* = 1) were revealed. All 9 OC and 1 BC Chechen patients with the *BRCA1* c.3629_3630delAG allele also carried linked c.1067G>A (Q356R; rs1799950) single nucleotide polymorphism (SNP). The population frequency of rs1799950 according to the gnomAD database is 0.05, while in the current study, it was observed in 11.5% of women of Chechen ethnicity. This SNP is not associated with pathogenic *BRCA1* variants in patients of Slavic origin (data not shown). Taken together, *BRCA1* Q356R is likely to be a benign substitution, being a passenger for *BRCA1* c.3629_3630delAG pathogenic variant. We investigated further whether this allele is located in *cis* or *trans* relative to the *BRCA1* c.3629_3630delAG. Five carcinomas were informative for the loss‐of‐heterozygosity analysis; all these tumors demonstrated concurrent loss of c.3629_3630delAG and c.1067G>A alleles indicating that they are located on the same chromosome. Therefore, *BRCA1* c.3629_3630delAG is a genuine founder allele but not a mutational hot spot.

**TABLE 2:** Pathogenic variants and variants of unknown significance observed in Chechen patients

Variant	dbSNP ID	ACMG classification	Num. of cases	Patient's characteristics	Described in other populations
*BRCA1*					
c.3629_3630delAG	rs80357589	Pathogenic	10	HGSOC, 59 HGSOC + BC, unspecified, 64 HGSOC, 34 HGSOC, 45 HGSOC, 53 HGSOC, 58 HGSOC, 51 HGSOC, 40 HGSOC, 64 BC, unspecified, 24	South of France^12^
c.5296delA	—	Pathogenic	2	HGSOC, 55 BC, triple‐negative, 36	Canada^13^
c.1338_1339delAG	—	Pathogenic	1	BC, triple‐negative, 55	No
c.5153‐2A>G [IVS18‐2A>G]	rs786202545	Pathogenic/likely pathogenic	1	BC, triple‐negative, 51	Not specified
c.3320A>G [E1107G]	rs1597864403	Uncertain significance	1	BC, triple‐negative, 49	Not specified
*BRCA2*					
c.9895C>T [Q3299X]	rs1555289997	Pathogenic	3	HGSOC, 61 HGSOC, 55 HGSOC, 58	Not specified
c.5351dupA	rs80359507	Pathogenic	1	HGSOC, 68	Argentina, Macedonia, Slovenia, Italy^14^, ^15^, ^16^, ^17^
c.7408_7409delTT	rs397507915	Pathogenic	1	HGSOC, 51	Not specified
c.9117G>A [P3039P]	rs28897756	Pathogenic	1	BC, receptor‐positive, 52	Japan, Korea, Latin Americans, Brazil^18^, ^19^, ^20^, ^21^
c.1714G>A [V572I]	rs587782713	Uncertain significance	1	HGSOC, 80	Colombia^22^
c.5860A>G [T1954A]	rs1566233345	Uncertain significance	1	HGSOC, 62	Not specified
*ATM*					
c.3511C>T [Q1171X]	rs876659067	Pathogenic	1	HGSOC, 53	France, China^23^, ^24^

*BRCA2* Q3299X is described in the ClinVar database as a pathogenic variant. Because it is located relatively close to the end of the coding portion of this gene, the evidence for its disease‐predisposing role is considered to be weak. To clarify the contribution of this variant to cancer predisposition, we examined the loss‐of‐heterozygosity (LOH) status at this locus in the tumor tissue and revealed somatic loss of the normal *BRCA2* allele in two out of three analyzed BCs. One HGSOC patient, who was negative for *BRCA1/2* germline mutations, was heterozygous for *ATM* pathogenic variant (c.3511C>T [Q1171X; rs876659067]) (Table 2). There were no women with *TP53* or *PALB2* germline alterations.

This study revealed a strong founder effect for *BRCA1* c.3629_3630delAG allele in Chechen BC and OC patients. *BRCA1* c.3629_3630delAG was firstly described in a French study.^12^ The analysis of large series of Russian BC and OC patients revealed one carrier of this variant in a woman with apparent Russian ethnicity.^26^ It is safe to conclude that currently available data do not demonstrate the elevated frequency of *BRCA1* c.3629_3630delAG allele in any of the studied populations, i.e., this variant appears to be characteristic specifically for Chechens. The detection of the founder mutations in Chechen patients is anticipated from historical and genetic studies.^5^, ^6^, ^27^, ^28^ Haplotyping analysis revealed a well‐preserved genetic identity of Chechens residing in various parts of the Caucasus mountains.^27^ Strong founder effect was demonstrated for patients with cystic fibrosis registered in the Chechen Republic.^28^ The identification of recurrent Chechen mutation *BRCA1* c.3629_3630delAG may have immediate clinical implication, as it allows non‐expensive screening of non‐selected BC and OC patients as well as healthy people with instances of BC or OC in their families. The analysis of founder pathogenic variants is significantly cheaper as compared to full‐length BRCA1/2 testing; therefore, this approach may therefore be more financially viable.

The analysis of consecutive or randomly selected HGSOC patients is apparently the most efficient approach for the analysis of the spectrum of *BRCA1/2* mutations in previously unstudied populations. However, it may cause some biases given that some mutations located within *BRCA1* and *BRCA2* genes demonstrate a preferential association with OC, while other pathogenic alleles tend to be linked to BC predisposition. Consequently, if the study is limited to HGSOC patients, some recurrent BC predisposing alleles may be missed. Notably, the *BRCA1* c.3629_3630delAG allele identified in the present study is located within the OC cluster region.^26^, ^29^

In conclusion, this small‐scale study identified recurrent *BRCA1* pathogenic variant characteristic for Chechen BC and OC patients. It also demonstrated the utility of HGSOC testing for rapid ethnicity‐specific mapping of the spectrum of *BRCA1/2* germline mutations.

## AUTHOR CONTRIBUTIONS

Conceptualization, Anna Sokolenko, Luisa Sultanova, and Evgeny Imyanitov; methodology, Anna Sokolenko, Ilya Stepanov, Alexandr Romanko, and Ilya Bizin; data collection and data curation, Aigul Venina, Tatiana Sokolova, Hedi Musayeva, Marina Tovgereeva, Mareta Magomedova, Khusein Akhmatkhanov, Elisa Vagapova, Elena Vasilyeva, Elvina Bakaeva, and Svetlana Aleksakhina; supervision, Elkhan Suleymanov, Svetlana Aleksakhina, and Evgeny Imyanitov; funding acquisition, Evgeny Imyanitov. All authors reviewed the manuscript and edited it for intellectual content and gave final approval for this version.

## FUNDING INFORMATION

The study was supported by the Russian Science Foundation [grant number 21–75‐30015].

## CONFLICT OF INTEREST

The authors have no conflict of interest.

## ETHICS STATEMENT

The study design was approved by the local Ethical Committee. All procedures performed in study were in accordance with the 1964 Helsinki declaration and its later amendments or comparable ethical standards.

## INFORMED CONSENT

All patients provided informed consent for the analysis of their clinical and molecular data.

## Data Availability

All data are available upon reasonable request.
